# Physiotherapy support for self-management of persisting musculoskeletal pain disorders

**DOI:** 10.4102/sajp.v77i1.1564

**Published:** 2021-10-29

**Authors:** Ina Diener

**Affiliations:** 1 Department of Physiotherapy, Faculty of Health Sciences, Stellenbosch University, Stellenbosch, South Africa

**Keywords:** self-management, self-management support, musculoskeletal pain, psychosocial barriers, behaviour change, psychologically informed physiotherapy

## Abstract

**Background:**

Musculoskeletal pain (MSKP) is an extremely common pain disorder in almost all populations. Self-management (SM) support is a programme to prepare people to self-manage their health condition effectively, while maintaining quality of life. SM is a cost-effective and context-specific strategy to address the global public health burden.

**Objectives:**

Self-management needs a change in behaviour from seeking unnecessary medical care to safely self-managing symptoms. As changing individuals’ behaviour is challenging, the objective of my literature review was to identify the characteristics, in both therapist and patient, to successfully engage in SM.

**Method:**

A narrative literature review, that could inform evidence-based support programmes for SM of MSKP.

**Results:**

Studies on successful implementation of SM of MSKP do not report strong outcomes. However, in more recent years a few positive outcomes were reported, possibly as a result of research evidence for the application of psychosocial skills and contemporary pain neuroscience in the management of persistent MSKP.

**Conclusion:**

Psychologically-informed physiotherapy, addressing psychosocial barriers to the maintenance of SM programmes, could facilitate more successful outcomes.

**Clinical implications:**

Before engaging in a SM support programme, obstacles to behaviour change must be identified and addressed in a SM support programme, to facilitate individuals towards taking safe responsibility for their healthcare. Therapists working with patients with persistent MSKP, should upskill themselves to be in line with the latest pain and psychosocial research literature. Moreover, communication skills training seems to be a priority for effective SM support programmes.

## Increasing cost and rising disability

Chronic musculoskeletal pain (MSKP) is a prevalent pain disorder in almost all populations, in low- and high-income countries, and is a leading cause of disability worldwide. Therefore, a narrative literature review was done to inform the introduction of self-management (SM) in healthcare for chronic MSKP. The Global Burden of Disease study in 2019 demonstrated that musculoskeletal disorders are amongst the 10 most important drivers of increasing burden, and globally there is growing disability as a result of MSKP conditions. The highest contribution to the need for rehabilitation comes from musculoskeletal disorders. Low back pain was the leading health condition contributing to the need for rehabilitation services in 134 out of the 204 countries analysed; and it is growing rapidly in low- and middle-income countries (Cieza et al. 2020). Epidemiological studies have highlighted that psychosocial factors are associated with MSKP and disability, and have informed us on the way in which these factors serve as prognostic indicators for the development of chronic pain. Findings indicate that several dimensions of patients’ illness perceptions are key predictors of pain, and are significantly associated with a more severe pain trajectory years later (Foster & Delitto 2011). Understanding the long-term trajectories of MSKP disorders should enable better management of the long-term course of persistent MSKP, and could form part of patient education for SM interventions.

Recent research outcomes demonstrate the need for a shift towards a biopsychosocial focus in healthcare (i.e. cognition and perceptions, and social context) which underlies the pain problem, rather than only focusing on a purely biomedical origin when treating patients with persistent MSKP (Foster & Delitto 2011; Lin et al. 2020). Persistent pain is a complex disorder where central and peripheral nociceptive processes are influenced by factors from multiple dimensions. The recent developments in pain science have changed the role of physiotherapy in MSKP disorders. Research on pain has largely converged in support of three concepts that have shifted the healthcare profession’s understanding and treatment of pain. Firstly, that pain is not a signal originating from bodily tissues; secondly, that pain is not an accurate measure of tissue injury; and thirdly, that the nervous system demonstrates plasticity, which then becomes a viable target of treatment (Parker & Madden 2020). Consistent recommendations for the management of persistent MSKP include, amongst others: patient-centred care; red flag and psychosocial factor screening; minimum radiological imaging; thorough first clinical examination; patient education; assessment and address of functional physical activity; and facilitating continuation or resumption of work (Lin et al. 2020).

Persistent MSKP has huge negative effects on people’s well-being and cost to society. Treatment outcomes for people with persistent MSKP are modest and related healthcare cost is high. Healthcare systems globally are facing great challenges being increasingly under pressure to provide financially unsustainable healthcare services to patients with persistent MSKP (Cieza et al. 2020; Foster & Delitto 2011). In considering how best to develop an effective system that delivers quality care and value for money, the role that patients play has become ever more important. Self-management is a safe, community-based and effective way for patients with persistent MSKP to manage pain and disability. Self-management support involves provision of interventions to prepare people to manage health conditions effectively and maintain quality of life, and should therefore identify obstacles to change of behaviour. It should also support individuals towards safely taking responsibility for their own healthcare.

## Self-management: The current state of evidence

Self-management is endorsed in most guidelines for the management of MSKP, but the outcomes from studies on SM are not promising. Several systematic literature reviews demonstrate that SM has no or marginal benefit for patients with MSKP disorders (Elbers et al. 2018; Oliveira et al. 2012a; Reid et al. 2008). A few systematic reviews on the effectiveness of SM suggest improvements in knowledge and self-efficacy, but small or negligible effects on function in comparison to usual care, wait-list, or attention-controls (Du et al. 2011; Kroon et al. 2014; Nolte & Osborne 2013). In an analysis of the results of several randomised controlled trials (RCTs), strong evidence was found that self-efficacy and depression at baseline predict outcome, and that pain catastrophising and physical activity can mediate the outcome of SM (Miles et al. 2011). Other potential explanations for weak results in SM studies mentioned by these authors include: sub-grouping of patients with particular characteristics may be more effective than generic SM programmes; a strong therapeutic relationship with the participant could facilitate a change in behaviour towards SM. It has, however, been demonstrated that lasting behaviour change is challenging (Webb & Sheeran 2006). Self-management support programmes should be designed to take these factors into account.

Recently, support programmes for SM have been developed to incorporate contemporary pain science and the recent evidence on how to best manage chronic MSKP with a combination of education and exercise. Some noteworthy examples are from Denmark, where a group of researchers formulated the ‘Good Life with Osteoarthritis (OA) in Denmark’ (GLA:D™) programme for SM support for people with knee and hip OA (Skou & Roos 2017). The GLA:D^TM^ programme for patients comprises a ‘minimal intervention’ with three sessions of patient education delivered over 2 weeks and 12 sessions of supervised neuromuscular exercise delivered twice weekly for 6 weeks. The patient education emphasised knowledge of OA and treatment of OA with a particular focus on exercise, its beneficial effects on symptoms and general health, and self-help advice. After the 8-week programme, the patients are encouraged to continue being physically active and to exercise. Individual strategies for the continuation of physical activity and exercise are discussed at the 3-month follow-up. After having participated in the GLA:D™ Knee and Hip’ programmes, patients’ pain decreased by 26% – 27%, function improved, fewer people took pain-killers and fewer people were on sick leave. The same group of researchers have now developed an evidence-based SM support programme for chronic low back pain, with the aim to improve pain beliefs and management skills (Kjaer et al. 2018). Patient education is provided in two 1-h lectures, and includes pain mechanisms, active coping strategies, imaging, physical activity and exercise. The exercise programme includes 16 supervised 1-h sessions over 8 weeks, with the aim to improve overall back fitness and, at the same time, encourage patients to explore variations in movement by incorporating education content into the exercise sessions. A pilot study demonstrated the programme feasibility for a nation-wide RCT, and secondary outcomes demonstrated better patient-reported outcomes compared to before the programme was implemented.

A 6-week ‘ChrOnic pain self-ManageMent with pain science EducatioN and exerCisE’ (COMMENCE) programme, resulted in improved function, pain intensity, pain knowledge, catastrophising, self-efficacy, satisfaction with health care, and increased ‘global rating of change’ scores over a 12-week follow-up period in comparison with usual care (Miller et al. 2020). The SM support programme included education about SM, pain science and cognitive behavioural principles to support behaviour change, combined with an exercise programme tailored to participants’ goals and abilities. The SM support programme led by trained physiotherapists, consisted of two visits per week over 6 weeks. One visit in a group format incorporated education about SM and pain science, and cognitive behavioural principles to support behaviour change. The second was individually tailored, aiming to support implementation of SM plans and the development of an exercise programme adapted to the participant’s goals and abilities. Participants were encouraged to continue SM beyond the end of the intervention up to measurement at 12 weeks follow-up. The COMMENCE programme was more effective than usual care at improving function, pain, catastrophic thinking, self-efficacy, pain knowledge, satisfaction, and perceived change, in a group of patients with chronic non-cancer pain.

It can be challenging to effectively integrate behavioural and cognitive components into the biopsychosocial management of MSKP. Depression and self-efficacy predicted outcome irrespective of intervention, suggesting that these should be targeted at early stages to prevent the transition to chronic disability (Miles et al. 2011; Webb & Sheeran 2006). This was confirmed by a recent literature review of the intervention components and patient outcomes of studies integrating these components in physiotherapy. The researchers’ aim was to match the interventions with a definition of behavioural medicine in physiotherapy, while categorising the behaviour change techniques used for patients with MSKP. The results demonstrated that the integrated psychosocial, behavioural, and biomedical or physical aspects applied in the reviewed interventions complied with the existing definition of behavioural medicine in physiotherapy. The frequently reported component was to improve self-efficacy. The long-term follow-ups mostly showed positive effects, for example, reduction of fear and catastrophising, change in pain beliefs, increase in activities and pain SM strategies, and improved stress management (Soderlund et al. 2020). The findings of the study suggest that factors like self-efficacy, depression, pain catastrophising and physical activity impact on outcomes of the SM programmes. There is evidence that understanding of pain neuroscience and early identification of psychological barriers, may improve these factors (Diener, Kargela & Louw 2016; Louw et al. 2016). Utilising these in the SM support programmes may thus improve the outcomes of these programmes.

## Support programmes to prepare individuals with persistent musculoskeletal pain for self-management

The SM support should prepare people to manage health conditions effectively and maintain quality of life. The preparatory programme should thus prepare and empower patients towards SM of their chronic MSKP disorders, and support them during the process (Cooper, Smith & Hancock 2009; Hutting et al. 2020; Peek et al. 2016). Psychological adjustment in resilience and preparedness to take responsibility may lead to favourable outcomes. For successful SM, a person with persistent MSKP must be able to manage the symptoms, treatment, physical and psychosocial consequences, and lifestyle changes inherent to living with a chronic condition. Ideally, a SM support programme should address all these factors, as newly learned behaviours can be difficult to maintain.

The outcome of a support programme for SM should be measured in terms of ‘enablement’ of the patient. As ‘enablement’ can mirror the results differently from traditionally used outcome measures, outcomes should be measured with questions such as: whether the programme has been successful in empowering and supporting patients to have strategies to regain power over their own lives, accept their current status, achieve better health and reach their personal goals. The Patient Enablement Instrument (PEI) has been found to have fair content validity, construct validity and internal consistency in a group of patients with chronic MSKP (Enthoven et al. 2019).

## What is likely needed for successful self-management of a musculoskeletal pain disorder?

### Tutors of self-management support programmes

Self-management and SM support are considered important to physiotherapists, for people with MSKP disorders, but the way physiotherapists address this in practice does not seem to be optimal (Miles et al. 2011). Many clinicians hold unhelpful beliefs themselves; while others feel ill-equipped to explore and target the patient’s unhelpful beliefs that drive the pain. As a result, clinicians may reinforce unhelpful beliefs, behaviours and resultant disability among the patients they train for SM (Caneiro, Bunzli & O’Sullivan 2020). Elbers et al. (2018) suggested three reasons for SM programmes not being successful: behavioural change is difficult; self-efficacy for SM is often lacking; and empowerment towards active and resilient coping needs to be included. Patients express a need for SM support, suggesting there is room for empowerment towards self-managing their condition (Cooper et al. 2009).

Effective implementation of SM support programmes thus depends partially on the skills of *service providers*, of which communication skills seem to be the most important, to understand the individual and the barriers that may be faced when embarking on SM of persistent MSKP. Self-management interventions need tailoring to individual barriers, facilitators and needs as these play an important role in realising positive effects. Healthcare providers, may require additional training on how to collaborate with their patients or clients in setting health goals and decision-making (Mann, LeFort & VanDenKerkhof 2013). Experts in the field identified four factors that should guide the SM support provided: change of mind-set; a patient-specific programme; managing uncertainty; and support from the therapist (Stenner et al. 2015).

Physiotherapists are in a good position to empower patients towards SM of MSKP, and are frequently considered as part of the rehabilitation team (Hutting et al. 2020; Soderlund et al. 2020). Results from some successful outcomes in studies on the management of persistent MSKP have informed us on what kind of therapy or therapist is needed in the support programme, for SM to be successful (Foster & Delitto 2011). Therapists’ own beliefs about MSKP and the best management thereof plays an important role in the way a SM support programme is structured (Caneiro et al. 2020). Healthcare providers therefore need to have knowledge of the current evidence-base of care for persistent pain (Lin et al 2020; Parker & Madden 2020). Also, they should strive to follow researched requirements to implement a successful SM support programme and facilitate adherence to the programme (Hutting et al. 2020; Peek et al. 2016:127–135). A holistic patient-centred approach by the healthcare provider is one comprised of the three roles of: *educator, partner* and *coach* (Solvang & Fougner 2016:591–602), suggesting that changing to SM, needs education of the patient in a strong therapeutic relationship, and awareness of the living conditions of the patient. To fulfil these three roles, a therapist involved in the preparatory support programme, needs certain attributes to facilitate a successful SM outcome.

#### Communication skills

Clinical communication skills and a strong therapeutic relationship may facilitate person-centred care, shared decision-making and goal setting. Listening and motivating clinical communication, as meaningful conversations, may encourage an active role for people under therapists’ care. Listening is therapy in itself; it demonstrates to the person that their ‘story’ is valuable (Diener et al. 2016). Understanding what is important to a person and insights into a person’s life is vital to ensure a positive outcome in SM. Exploring the psychosocial context of the person in pain includes their expectations, existing depression and anxiety factors, and their social support system (Miller et al. 2020). Good communication between health providers and patients, and valuing patient autonomy can promote adherence and resultantly improve outcomes (Oliveira et al. 2012b). Motivational interviewing (MI) aims to strengthen personal commitment by respecting the individual’s autonomy and assisting them to reach a specific goal by exploring personal intentions or reasons for change (Alperstein & Sharpe 2016). Motivational interviewing is valuable to facilitate peoples’ desire to help themselves and to facilitate a behavioural change. Patient education and communication strategies such as pain neuroscience education and MI have been developed for the management of persistent disabling pain. (Nijs et al. 2020). Training healthcare providers in clinical communication skills can enhance patient outcomes; and this needs to be a priority for the providers of the SM support programmes.

A strong therapeutic relationship is built on trust and mutual respect and is dependent on effective clinical communication skills. In a qualitative study, participants made it clear that therapeutic relationships do not ‘just happen’. Clinicians need to be *present* (in-the-moment), *receptive* (an open attitude and a focused receptivity), *genuine* (being yourself, and being honest), and *committed* (be engaged in your role). The therapeutic relationship between patient and provider is considered a central component of patient-centred care and patient engagement, leading to patient satisfaction and adherence to treatment (Miciak et al. 2018). Patient-centred care emphasises equal partnerships between people involved in planning, developing and accessing care to ensure it meets the person’s needs. An overwhelming amount of evidence supporting a person-centred approach has placed it at the core of healthcare for people living with long term conditions (Cooper et al. 2009; Lin et al. 2020; Miciak et al. 2018; Nijs et al. 2020; Peek et al. 2016). Clinicians need to have skills and knowledge in both evidence-based practice and communication, so that both evidence and patient preferences can be incorporated into consultations (Nijs et al. 2020).

#### Educational skills

Firstly, clinicians’ awareness and knowledge of psychosocial contributions to persistent pain is important for support and education of the patient. Screening for recommended psychosocial risk factors (aka yellow flags), which may be barriers to recovery has been researched abundantly in recent years. It has become apparent that these psychosocial risk factors, like fear of movement, anxiety, maladaptive beliefs, etc., may in many cases be the dominating factors associated with recovery in MSKP disorders, and need to be identified and addressed (Diener et al. 2016; Foster & Delitto 2011; Lin et al. 2020; Parker & Madden 2020). The role of vulnerability factors such as pain-related fear, catastrophising, and avoidance have been well-documented in the development and maintenance of chronic pain disability (Meulders 2019). Caneiro et al. (2020) encouraged clinicians to exercise self-reflection to explore their own beliefs and better understand their biases, which may influence their management of patients with MSKP. They are of the opinion that disconfirming unhelpful beliefs through behavioural learning, self-reflection and evidence-based education, can promote a new understanding that empowers SM.

Secondly, health literacy should form part of education of the patient. Health literacy describes the personal skills and environmental conditions that enable individuals to obtain, understand, and use information to make decisions and take actions that will have an impact on their health status (Muscat et al. 2020). Being knowledgeable about health issues could facilitate shared decision-making. Even though shared decision-making has not been tested for its role in the outcome of management of chronic MSKP conditions, there is a potential benefit in a patient-centred care approach, as it gives a voice to individuals and allows them more control towards the healthcare they choose to receive, and their own participation (Tousignant-Laflamme et al. 2017). Health education supports the patient’s thought processes and decision-making. This information should, however, always follow the principles of contemporary pain neuroscience, to not increase pain related fear (Louw et al. 2016). Physiotherapists providing care for people presenting with musculoskeletal conditions are in the privileged position of being able to promote healthy behaviour (Lewis et al. 2021) by providing evidence-based education on:

- the natural course of MSKP conditions (Foster & Delitto 2011; Lin et al. 2020; Parker & Madden 2020)
- the evidence-base for radiological imaging and the negative effect of unnecessary scanning (Lin et al. 2020; Caneiro et al. 2020)
- the benefits of activity and exercise (Elbers et al. 2018; Kjaer et al. 2018; Lin et al. 2020; Miller et al. 2020; Skou & Roos 2017; Wade 2020).

The third part of education is therapeutic neuroscience education (TNE), that has been demonstrated to help patients to reconceptualise their pain, leading to reduction in pain, disability, psychosocial factors and healthcare utilisation, while improving movement and function (Louw et al. 2016). The aim of the TNE is to guide patients’ pain beliefs and perceptions to facilitate the acquisition of adaptive pain-coping strategies (Diener et al. 2016; Nijs et al. 2020).

#### Physical examination and clinical reasoning skills

The clinician, embarking on a SM support programme for a patient, should have the knowledge and skill to do an appropriate musculoskeletal physical examination, to rule out red flags and confirm the pain type the patient is complaining of. Clinical reasoning based on a thorough interview and physical examination should differentiate nociceptive versus neuropathic versus nociplastic pain, which each needs a specific SM support approach. Whereas nociceptive and neuropathic pain may need advice on positions and movements, SM support for nociplastic pain should care for the psychosocial factors driving the pain experience. Pain should never be accepted as persistent pain with no apparent physical reason before a thorough questioning and physical examination have cleared all red flags and confirmed the type of pain.

#### Physical rehabilitation skills

Rehabilitation is about helping people recover from illness to return to what matters in life. The end-goal of rehabilitation is to optimise a patient’s self-rated quality of life and degree of social integration through optimising independence in activities, minimising pain and distress, and optimising the ability to adapt and respond to changes in circumstances (Wade 2020). Effective rehabilitation is a person-centred process, tailored to the individual patient’s needs, and importantly, personalised monitoring of changes associated with intervention. However, patients are often not adherent to rehabilitation interventions, and non-adherence can compromise the gains seen from an effective SM support programme. A strong therapeutic relationship, shared decision-making and patient-centred clinical communication may enhance adherence and patient outcomes. The SM support programme must therefore, for each individual patient, use a collection of interventions to meet the patient’s specific needs and evaluate the patient’s rehabilitation programme on a planned, ongoing basis, using simple targeted measures at appropriate intervals. By promoting behavioural change (Katz, Patterson & Zacharias 2019) and empowering participants to take control (Zuercher-Huerlimann et al. 2019), rehabilitation programmes may be maintained by participants. Physiotherapists need to ask themselves if they understand the importance of behavioural change and if they have the skills and knowledge to incorporate behavioural change as an integral part of the rehabilitation they offer (Lewis et al. 2021). Table 1 is a summary of the attributes therapists engaging in SM programmes should strive to master.

**TABLE 1:** Attributes of therapists supporting patients with musculoskeletal pain disorders towards SM.

Attributes of the therapist engaging in a SM support programme	Components	Reading
Communication skills	Listening and reflecting	Diener et al. (2016); Miciak et al. (2018); Nijs et al. (2020)
	Therapeutic alliance	
	Motivational interviewing	
Educational skills	Addressing psychosocial barriers to engaging in a SM programme	Meulders (2019); Caneiro et al. (2020)
	Self-reflection on own beliefs	
	Health education	
	Pain neuroscience education	
Physical rehabilitation skills	Person-centred	Wade (2020)
	Needs-tailored	
	Aspects to improve adherence	

SM, self-management.

### Participants of self-management programmes

In preparing for SM, clinicians should focus on important SM skills that *participants* possess. It is therefore important during the support programme, to assess participants’ skills and knowledge of SM, and their confidence in managing their own health. Factors such as readiness for change, health locus of control (HLOC), patient activation level of health, self-efficacy and pain beliefs need to be assessed and addressed during the support programme with training, to facilitate a successful transition to SM (Martin et al. 2019). Showing willingness to engage in SM of their persisting MSKP is in principle a mind-set and behaviour change. Therefore, behavioural counselling may help to increase their adherence to the programme, and boosting self-efficacy may facilitate a positive lifestyle change (Lewis et al. 2021). The SM support programmes should include assessment and training of the skills required for day-to-day management of chronic pain conditions, to ensure that individual barriers to SM are overcome and it succeeds.

#### Readiness for behaviour change

Patients included in a SM programme before they are ready to take responsibility, may fail to change their behaviour and thus not succeed The study of Katz et al. (2019) demonstrated the importance of a potential pre-programme targeted intervention for better patient engagement in an interdisciplinary pain programme. The ‘Pain Stages of Change’ (pre-contemplation, contemplation, action, and maintenance) Questionnaire is a useful tool to help identify how ready a patient is for behaviour change towards SM of their MSKP.

#### Health locus of control

The need for SM is individual-specific and may change over time. The HLOC refers to the extent to which individuals believe they can control events affecting their MSKP and other health conditions (Grotz et al. 2011) A person’s ‘locus’ is conceptualised as either internal or external. The internal locus refers to the extent to which a person believes they can influence their own life situation. An external locus refers to their belief that their decisions and life are beyond their own influence, decided by environmental factors which they cannot influence, or that decisions and life are determined by chance or fate. Higher levels of internal control are related to better SM competency (Zuercher-Huerlimann et al. 2019). The HLOC needs to be addressed in a preparatory programme for SM, to build confidence in the ability to exert control over motivation and behaviour.

#### Pain beliefs and self-efficacy

There are several maladaptive beliefs among individuals with a persistent MSKP. An 8-week interdisciplinary pain programme, associated with improvements in patient-related outcomes, demonstrated that fear of pain or re-injury was predictive of a patient being in the pre-contemplative stage of change, whereas pain self-efficacy was predictive of being in the contemplative, action, or maintenance stage of change (Katz et al. 2019). Illness beliefs, fear, expectation and perception of physical capacity influence self-efficacy (Caniero et al. 2020). Fear of movement is one of the most important reasons for the development of chronic pain-related disability (Meulders 2019). It is therefore important to have targeted interventions to decrease fear of pain or re-injury, and to enhance self-efficacy for better patient engagement and compliance. Believing that an injury or dysfunction is the reason for a patient’s pain and fear of movement-related pain, should be addressed promptly, as this can delay patients taking responsibility for SM. Radiological imaging results, biomedical explanations for their pain by medical practitioners, and internet information may all fuel maladaptive fear of pain or re-injury (Caneiro et al. 2020; Lin et al. 2020). Fearful participants may also need more support after they have started with SM, as flair-up episodes of pain may let them sink back into their mal-beliefs. Table 2 is a summary of the attributes participants engaging in SM programmes need.

**TABLE 2:** Attributes that participants most likely need to engage in successful self-management.

Attributes of the patient engaging in a SM support programme	Assessment	Reading
Readiness for behaviour change	Pain Stages of Change Questionnaire	Kerns (1997)
HLOC	HLOC	Wallston, Kaplan and Maides (1976)
Pain beliefs and self-efficacy	Pain Catastrophising Scale Tampa Scale for Kinesiophobiae11 Pain Self-Efficacy Questionnaire Patient Health Questionnaire	Sullivan, Bishop and Pivik (1995); Tkachuk and Harris (2012); Nicholas (2007); Kroenke, Spitzer and Williams (2001)

*SM, self-management; HLOC, Health locus of control*

## Conclusion

Epidemiological research demonstrates that persistent MSKP disorders pose a significant worldwide epidemiologic burden, displaying an escalating trend. There is an increasing recognition of the merits of ‘de-medicalising’ musculoskeletal care and shifting towards models of care that are more patient-centred and focused on supporting SM. The SM support programmes should in essence prepare participants for behaviour change, but should also be in place to provide support during the SM period. Self-management, a common component in interdisciplinary pain management programmes, is expected to facilitate more active and resilient coping. Healthcare providers working with patients with persistent MSKP should upskill to be in line with the latest pain neurophysiology and pain-related psychosocial research literature. Moreover, communication skills training of therapists needs to be a priority for effective SM support programmes. Research into SM support programmes for MSKP not only need to include the best treatment and rehabilitation techniques, but also assessment and training of psychosocial components that will facilitate and support behaviour change and compliance to SM. Although there are several global initiatives to address the global burden of persistent pain as a public health problem, there is a need to identify cost-effective and context-specific strategies for managing persistent MSKP disorders, to reduce the consequences of the current burden. Self-management support programmes are seen as one of the possibilities. Clinicians have a collective responsibility to educate patients, the community, funders, policymakers and other clinicians on safe and effective SM of MSKP, to help reduce the disability and cost burden in society, especially in a country like South Africa, where the disability burden is high and health resources are limited.
